# Push-Out Bond Strength Evaluation of Fiber-Reinforced Composite Resin Post Cemented with Self-Adhesive Resin Cement Using Different Adhesive Bonding Systems

**DOI:** 10.3390/ma14133639

**Published:** 2021-06-29

**Authors:** Yoon Lee, Junghyun Kim, Yooseok Shin

**Affiliations:** 1 Department of Conservative Dentistry, College of Dentistry, Gangneung Wonju National University, Gangneung 25457, Korea; yoonlee@gwnu.ac.kr; 2 Department of Conservative Dentistry, Wonju Severance Christian Hospital, Wonju College of Medicine, Yonsei University, Wonju 26426, Korea; tanya900@gmail.com; 3 Oral Science Research Center, Department of Conservative Dentistry, College of Dentistry, Yonsei University, Seoul 03722, Korea

**Keywords:** universal adhesive, self-adhesive cement, push-out bond strength, fiber-reinforced resin post

## Abstract

The purpose of this in vitro study was to evaluate the push-out bond strength of fiber-reinforced resin posts using self-adhesive cements with different adhesive systems. A total of 50 single-rooted human maxillary premolars with fully developed apices and 15–16 mm straight root canals were selected. The teeth were divided into 10 groups with coronal and apical parts according to the adhesive bonding system and luting material used: one universal adhesive with MDP-containing self-adhesive resin cement; another universal adhesive with MDP-containing self-adhesive resin cement; universal primer with MDP-containing self-adhesive resin cement; universal primer with dual-cure resin cement; MDP-containing self-adhesive resin cement only (Control). Each specimen was subjected to a fatigue load of 600,000 cycles using a chewing simulator with sliding movement and cut horizontally for push-out bond strength testing. Statistical evaluation consisted of a one-way ANOVA test using SPSS v23.0. The highest bond strength (7.05 MPa) was obtained in the coronal part of the Single Bond universal group treated with MDP-containing self-adhesive resin cement and the lowest strength (4.77 MPa) was observed in apical part of MDP-containing self-adhesive resin cement group (Control). However, the one-way ANOVA results showed no significant difference between all 10 groups (*p* > 0.05). The self-adhesive cement without adhesive bonding showed no statistically different value compared to self-adhesive cements with adhesive bonding.

## 1. Introduction

Selection of proper adhesives, luting materials, and procedures is important for post-retained restorations [1]. Because the bonding of cement materials with conventional adhesives remains a challenge, bonding posts to root canal dentin requires consideration of tooth structures and understanding of the applied adhesive system [2,3]. When using light-curing adhesive systems, the quality of polymerization has been questioned because light penetration to reach inside the root is not guaranteed [4,5]. Therefore, many dentists use dual-cure adhesive or self-cure adhesive systems where the light quality does not affect the polymerization quality. However, these dual-cure and self-cure systems have significant drawbacks. When using dual-cure resin-based cements, the acidic nature of the adhesives protonate the amine component of peroxide-amine initiator systems [6]. Therefore, a retarding effect was observed in the curing performance of the cement, prohibiting ideal interactions between the adhesive and cement, preventing complete polymerization. Self-adhesive resin cements (SARCs) have been recently developed and, according to the manufacturers, SARCs do not require tooth surface pretreatment, which significantly simplifies the clinical implementation and possibly prevents side effects caused by pre-applied adhesives on dentin.

However, contradictory results regarding the bond strength of SARCs compared to conventional adhesives have been reported [7,8,9,10]. Ferracane [11] and Bitter et al. [12] showed that RelyX U100 (SARC, 3M ESPE, St. Paul, MN, USA, a former version of RelyX U200) only superficially interacts with root dentin and the adhesives penetrated just a few µm inside the dentinal tubules. Previous studies have reported that using a dual-polymerizing resin adhesive pretreatment for SARC is a beneficial technique for bonding post systems [11,12,13,14,15]. In contrast, Pegorara and Attart showed that SARCs alone can bond to root dentin and restorative materials, exhibiting high bond strength and low solubility compared to conventional adhesive techniques [16,17].

This study was performed to compare the post bond strength when SARCs are used alone or with light- or self-cured adhesives. All-Bond Universal (ABU) and Single Bond Universal (SBU), which are commonly used adhesives, are known to inhibit the polymerization of SARC due to their low pH (>3 and >2 for ABU and SBU, respectively) [18]. Both adhesives are mildly acidic, which may have a significant impact on cement polymerization compared to chemical-type adhesives. When the surface of the adhesive layer is contacted with oxygen, incomplete polymerization may occur, and when acidic monomers come in direct contact with the cement, additional polymerization problems may occur. In addition, when light curing adhesives are used, the curing light may not penetrate the root during curing. Thus, researchers have claimed that chemical-type adhesives are superior for post bonding to achieve higher bond strength.

The bond strength of the posts can be analyzed via microtensile testing, specifically push-out and pull-out tests. Push-out bond tests are considered to be more accurate, reliable, and appropriate for measuring the bond strength of the post [3,19]. Moreover, the push-out bond tests simulate clinical situations more closely [20]. The clinical success of a post-and-core restoration depends on post retention, as debonding is the most common cause of failure for fiber-reinforced posts [21,22]. Moreover, proper adhesion at the post-resin interface is important for distributing the stress generated during occlusion [23,24].

Therefore, the purpose of this study was to evaluate the push-out bond strength of fiber-reinforced posts after application of self-adhesive cements with different adhesive systems. The null hypothesis was that there is no difference in push-out bond strengths of fiber-reinforced resin post when bonded with either chemical-type adhesives or light curing adhesives in combination with SARCs.

## 2. Materials and Methods

The study design was approved by the institutional review board of the Yonsei University Wonju College of Medicine (number CR317310). A total of 50 single-rooted human maxillary premolars with fully developed apices were selected for this study. The teeth were recently extracted for orthodontic or periodontal reasons and had 15–16 mm straight root canals. The exclusion criteria were previous endodontic treatments and the presence of root cracks or root caries. Teeth were hand-scaled and stored in saline at 4 °C for a maximum of 6 months before use. The main outcome was the determination of push-out bond strength and the experimental unit was the roots embedded in self-cured resin. The materials used in this study are shown in Table 1.

**Figure 1:**
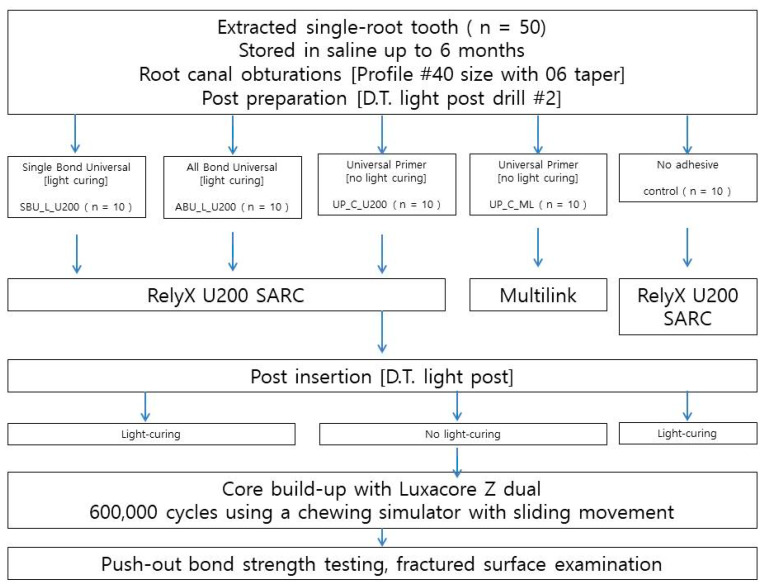
Flowchart of the cementation procedure for the specimens.

The protocols used in this study are schematically shown in Figure 1. The crown of each tooth was removed 1–2 mm coronal to the cement-enamel junction with a slow-speed diamond bur under running water. The working length was set to 1 mm above the anatomic apex to standardize 13 mm of working length from the cement-enamel junction. Root canals were performed using ProFile NiTi rotary instruments (Dentsply Maillefer, Tulsa, OK, USA) with up to #40 size with a 06 taper. Root canals were irrigated with 2 mL of 5.25% sodium hypochlorite solution between each instrument change. The canals were dried using size 30 paper points (Dentsply Maillefer, Tulsa, OK, USA). Subsequently, root canals were obturated by gutta-percha with a resin-based sealer (AH plus; Dentsply Maillefer, Tulsa, OK, USA) using the continuous wave compaction technique.

A total of 50 roots were randomly divided into 5 groups (n = 10) after the endodontic procedure with 8 one-canaled teeth and 2 two-canaled teeth in each group and stored in saline for 24 h. Next, posts were cemented to each group containing 10 roots, using 5 different post luting procedures with either RelyX U200 (3M ESPE, St. Paul, MN, USA) or Multilink (Bisco, Schaumburg, IL, USA).

For the Single Bond Universal (SBU_L_U200) group, the gutta-percha in the coronal part of the tooth was removed using #2 and #3 Gates Glidden drills. The post space was prepared at a length of 8 mm using a DT2 Light-Post preparation drill with a diameter of 1.5 mm. It was then etched with 37% phosphoric acid for 15 s, washed with water, and dried. Single Bond Universal (3M ESPE, St. Paul, MN, USA) was applied to the canal surface followed by light-polymerization at 1000 mW/cm^2^ (VALO light irradiator; Ultradent Products Inc., South Jordan, UT, USA). Afterwards, RelyX U200 was applied to the root canal according to the manufacturer’s instructions. Using needle tubes, the cement was loaded into the canal, filling it from bottom to top and spread onto the D.T. light-post (Bisco, Schaumburg, IL, USA) before insertion. The root canals were light-polymerized for 40 s through the posts.

For the All-Bond Universal (ABU_L_U200) group, the canals were prepared in a similar manner as that used for the SBU_L_U200 group. After All-Bond Universal was applied to the canals, the adhesives were light-polymerized for 15 s. A D.T. Light-post with RelyX U200 was loaded into the canal and light-polymerized for 40 s in the same manner.

For the universal primer (UP_C_U200) group, the post spaces were prepared as described for the SBU_L_U200 group. The universal primer was applied to the canal and the D.T. Light-post with RelyX U200 was inserted in same manner as described above.

For the universal primer with Multilink (UP_C_ML) group, Multilink cement was applied instead of RelyX U200 in the same manner as the above three groups and light-polymerization was not performed.

For the control group, no adhesives were applied and only the RelyX U200 (Control) cement was applied with post-insertion.

With 2-canal roots, both canals were endodontically obturated in the same manner, and the straighter and wider canal was chosen as the main canal. A single operator performed all procedures.

A total of 50 roots were core built-up with LuxaCore Z Dual (DMG, Hamburg, Germany) to a height of 3–4 mm at the coronal area. The specimens were embedded in a self-polymerizing acrylic resin (Jet Tooth Shade, Lang Dental Manufacturing Co., Wheeling, IL, USA) up to 1 mm apical from the cement-enamel junction. Each specimen was subjected to a fatigue load of 600,000 cycles using a chewing simulator with sliding movement (CS-4.8, SD Mechatronik, Feldkirchen-Westerham, Germany), which simulated 1 year of function. The chewing simulator applied a mechanical loading (50 N, 1.6 Hz) and thermal aging cycling between 5 and 55 °C 1263 times.

**Figure 2:**
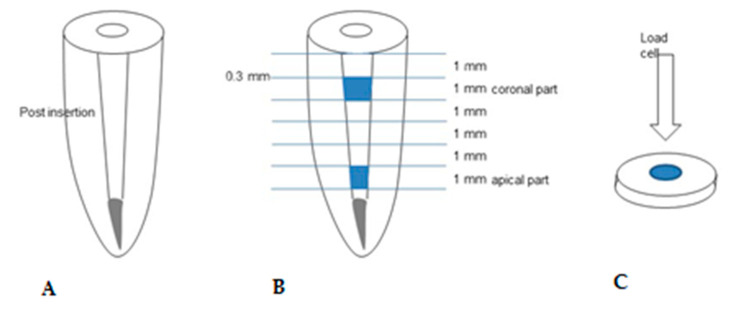
(**A**) Specimen preparation for push-out bond strength. (**B**) Specimen preparation for push-out bond strength test. Specimens were sectioned horizontally into 1-mm-thick slices, with a 0.3-mm-thick saw. (**C**) Fiber-reinforced composite resin post positioned in the center of the post space and cemented using different adhesive systems.

The specimens were sectioned horizontally into 1-mm-thick slices using a low-speed diamond saw (Met-Saw; R&B Co Ltd.) under water cooling, as shown in Figure 2. The coronal and apical parts of the teeth were tested to evaluate the push-out bond strength. The push-out bond strength test was performed using a universal testing machine (EZ-S; Shimadzu Scientific Instruments, Kyoto, Japan). The load was directed from the apical to coronal direction at a cross-head speed of 0.5 mm/s until bond failure occurred. The debonding force value (N) was divided by the post-dentin surface area to calculate the push-out bond strength (MPa). The total bonding area was calculated as follows:

$$\pi \left[R+r\right]\left[h^{2}+{\left(R-r\right)}^{2}\right]0.5$$

where R is the post radius, r is the apical post radius, and h is the slice thickness.

The average debonding force and push-out bond strength of all 10 groups were analyzed using two-way analysis of variance using a statistical software package (SPSS v23.0, SPSS Corp., Chicago, IL, USA) with adhesive type and SARC as independent factors at a significance level of 0.05. In addition, one-way analysis of variance was used to compare the means among all 10 groups.

A total of 10 fiber-reinforced posts from the coronal and apical parts of all groups were examined with SEM imaging to study the surface morphological characteristics of the failure mode. Specimens were treated with gold sputtering before SEM evaluation.

## 3. Results

The results of the push-out bond strength tests are summarized in Table 2. The highest bond strengths (7.05 MPa) were observed in the coronal part of the Single Bond universal group with RelyX U200 (SBU_L_U200 group) and the lowest strengths (4.77 MPa) were found for the apical part of the RelyX U200 group (Control). The results of the two-way ANOVA showed that the adhesive type had a significant effect on the push-out bond strength while the SARC type did not have a significant effect. (Table 3). However, the one-way ANOVA results showed no significant difference among all 10 groups.

The most frequent mode of failure was adhesive failure between the dentin and luting material. Single Bond universal bonding (SBU_L_U200) showed a higher frequency of mixed failure than that of the control group. In addition, cohesive failure of the post was observed only in the SBU_L_U200 and UP_C_U200 groups, indicating that the bonding strength of the two groups could be superior to those of the other groups (Table 4).

**Figure 3:**
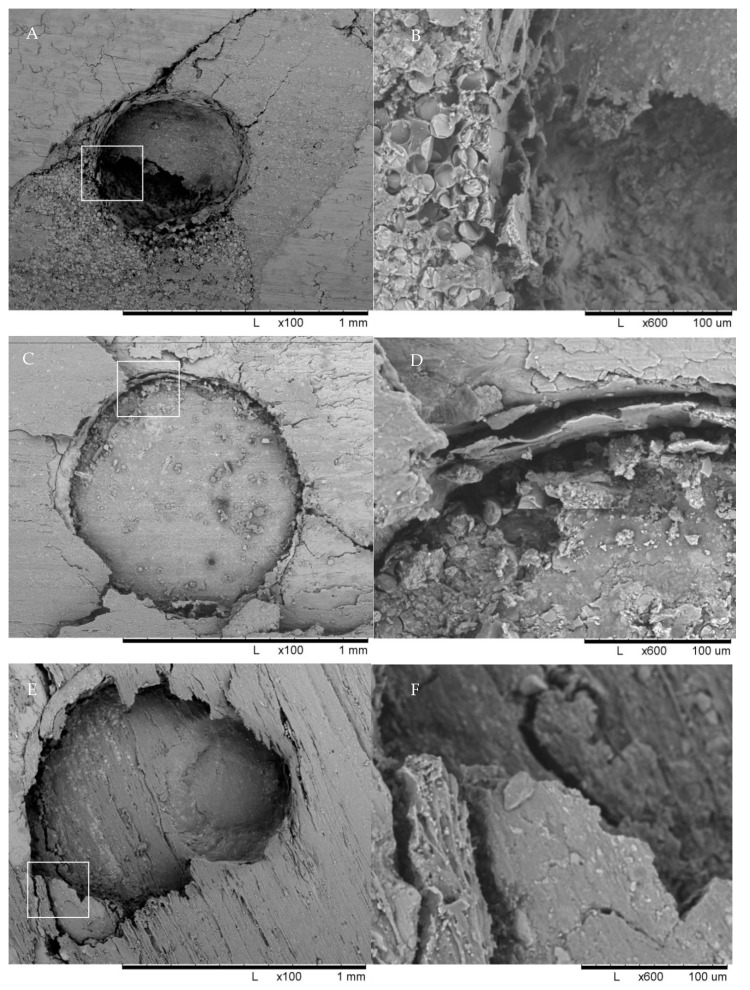

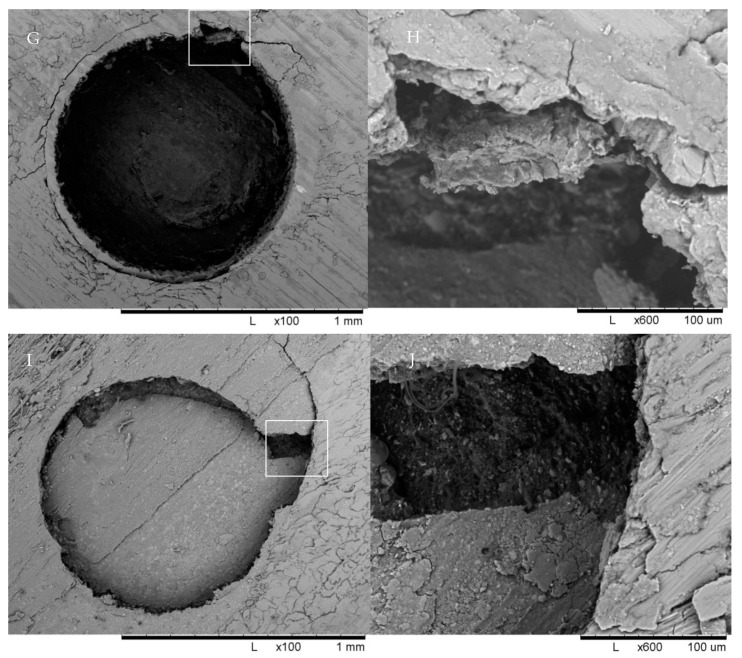
SEM images of specimens from selected groups. Cohesive, adhesive, and mixed failures of the posts can be seen. Single Bond group (SBU_L_U200) cohesive failure of posts (**A**,**B**). All-Bond group (ABU_L_U200) adhesive failure of the luting materials (**C**,**D**). Universal primer group (UP_C_U200) mixed failure of posts and luting materials (**E**,**F**). Universal primer with multilink group (UP_C_ML) adhesive failure of the luting materials (**G**,**H**). Control group (Control) adhesive failure of the luting materials (**I**,**J**).

SEM images from selected groups are shown in Figure 3.

## 4. Discussion

The use of SARC resulted in statistically significant difference according to adhesive type, i.e., light curing, chemical curing, and self-adhesive groups, in terms of push-out bond strength according to two-way ANOVA. Therefore the null hypothesis was rejected. For the light-cured group (SBU_L_U200, ABU_L_U200), a decreasing trend in bond strength from the coronal to apical parts was observed. The coronal part of the Single Bond universal light-cured group (SBU_L) showed the best values, though the difference was not statistically significant. The control group, where the post was bonded without adhesive, showed lower strengths than the mean of the other groups, consistent with the results of previous studies [10].

The bond strength of the Single Bond universal group (SBU_L_U200) was particularly interesting. Although it is a light-cured adhesive, the apical part showed higher bond strength than that of the apical part of the conventional chemical adhesive group. Single Bond universal is known to self-cure by reacting with components in self-adhesive U200 cement. The results presented herein indicate that that dual-curing is possible in the apical part, which may increase the bond strength. In addition, a clinically acceptable bonding strength was obtained even in groups without adhesive bonding (Control). Considering that this experiment simulated more than 1 year of use with chewing simulation (600,000 cycles) [25], the results are likely to be clinically relevant.

This study showed similar results compared to the relevant literature of light-cured adhesives.[10] The bond strength of the apical part was lower than that of the coronal part, which is likely due to the increased distance from the light source. It is also known that the number of dentinal tubules in the apical part is relatively small compared to that of the coronal section, which may reduce the bond strength in the apical portions [26]. However, in this study, the difference between the two regions was not statistically significant.

The push-out bond strengths measured herein were lower compared to a previous study by Barreto et al. who obtained higher values of approximately 11.5 MPa [27]. This may be the due to aging with 600,000 cycles of chewing, which reproduces more than 1 year of typical use, which was not performed in the previous study. Sodium hypochlorite was used in accordance with the Rely-X U200 manufacturers’ instructions, which may cause decreased bonding strength compared to saline irrigation [27].

For the light-cured adhesives, the apical part of the post may not be adequately light cured due to insufficient irradiation. On the hand, chemical type adhesives are not limited by the amount of irradiatio**n.** A previous study also reported a difference in post bond strength between the apical and coronal parts. However, it is known that the D.T. light post used in this experiment transmits light more effectively through the post compared to other fiber posts [28]. That may explain why there was not any significant difference among groups according to one-way analysis of variance.

Clinically, it was difficult to reach the apical portion with light irradiation since the gingiva and bone obstructed the tooth. However, the in vitro study may have transmitted the light more effectively during the experiment. Since the experiment was performed after sectioning the crown of the extracted tooth, the oral structures which may interfere with irradiation from the light source were reduced compared to the in vivo environment. That may also explain why there was not any significant difference among groups according to one-way analysis of variance.

The most frequent mode of failure observed in this study was adhesive failure between the dentin and luting material. In addition, more mixed failures were observed in the SBU_L_U200 group, as confirmed by the SEM images. The cause of high adhesive failure rate with post may be due to the lack of hydrofluoric acid or silane treatment.

This study showed that the light-cured adhesive is generally weaker in the apical part than in the coronal part, but the difference was not statistically significant, especially in the Single Bond universal group (SBU_L_U200). In addition, the control group with SARC and without adhesive bonding, showed clinically acceptable push-bond strength. SARC can be used to minimize the number of clinical steps, which may reduce the clinical errors associated with the bonding process. Further in vivo studies are needed to verify these in vitro results.

## 5. Conclusions

Within the limitations of this in vitro study, the following conclusions can be drawn: the adhesive type has a significant effect on push-out bond strength when fiber reinforced resin posts are bonded with SARC; SARC without adhesive bonding showed no statistically different bonding strengths compared to that of the SARC with adhesive bonding, and SARC without any adhesive bonding may be considered as a clinical alternative to SARCs with adhesive bondings.

## Figures and Tables

**Table 1:** Composition of the materials used.

Materials (Lot number)	Composition	Manufacturer
RelyX U200 (651113)	Base: methacrylate monomers containing acid groups, methacrylate monomers, silanated fillers, initiator components, and stabilizer Catalyst: methacrylate monomer, alkaline fillers, silanated fillers, initiator components	3M ESPE St. Paul, MN, USA
Multilink^®^ N (T21230)	DMA, HEMA, Ba-glass filler, ytterbium fluoride, spheroid mixed oxide, and phosphoric acid acrylate	Ivoclar Vivadent, Schaan, Liechtenstein
Single Bond Universal (648291)	MDP, Bis-GMA, HEMA, decamethylene DMA, ethanol, water, silane treated silica, 2-propionic acid, -methyl-, reaction products with 1,10-decanediol and phosphorous oxide, copolymer of acrylic and itaconic acid, dimethylaminobenzoate(-4), camphorquinone, (dimethylamino)ethyl methacrylate, and methyl ethyl ketone	3M ESPE, St. Paul, MN, USA
ALL-BOND Universal (1700002858)	MDP, Bis-GMA, HEMA, ethanol, water, and initiators	Bisco, Schaumburg, IL, USA
Universal Primer (1700002447, 1700002448)	MDP, Bis-GMA, HEMA, ethanol, water, and initiators	Bisco, Schaumburg, IL, USA

HEMA: 2-hydroxyethyl methacrylate; MDP: methacryloyloxydecyldihydrogen phosphate; DMA: dimethacrylate; bis-GMA: bisphenol A-glycidyl methacrylate.

**Table 2:** Comparison of the debonding force with push-out bond strength of the fiber-reinforced composite posts according to treatment with adhesive primers.

Variable	SBU_L_U200		ABU_L_U200		UP_C_U200		UP_C_ML		Control		
	Apical	Coronal	Apical	Coronal	Apical	Coronal	Apical	Coronal	Apical	Coronal	*p*
force (N)	20.11	27.09	17.00	23.05	19.13	24.43	19.06	22.33	15.71	18.33	0.31
Push-out bond strength (MPa)	6.21	7.05	5.25	5.99	5.91	6.36	5.89	5.81	4.85	4.77	0.55

**Table 3:** Results of the two ANOVA analysis.

Dependent Variable: Pushout Bond Strength					
Source	Type III Sum of Squares	df	Mean Square	F	Sig.
Corrected Model	33.671 ^a^	3	11.224	2.191	0.094
Intercept	2345.293	1	2345.293	457.766	0.000
adhesive	33.631	2	16.816	3.282	0.042
cement	0.012	1	0.012	0.002	0.962
adhesive * cement	0.000	0	–	–	–
Error	491.842	96	5.123	–	–
Total	3901.257	100	–	–	–
Corrected Total	525.512	99	–	–	–

^a^ R-Squared = 0.064 (Adjusted R-Squared = 0.035).

**Table 4:** Fracture modes at the coronal and apical parts in each group.

Type of Fracture		Fracture Mode				
		Adhesive (Post)	Adhesive (Dentin)	Cohesive (Post)	Cohesive (Luting)	Mixed
SBU_L_U200	Coronal	4	3	1	–	2
	Apical	3	3	–	–	4
ABU_L_U200	Coronal	4	4	–	–	2
	Apical	5	3	–	–	2
UP_C_U200	Coronal	4	3	1	–	2
	Apical	5	3	–	–	2
UP_C_ML	Coronal	6	1	–	–	3
	Apical	7	2	–	–	1
Control	Coronal	6	3	–	–	1
	Apical	7	1	–	–	2
Total		51	26	2	–	21

## Data Availability

Not applicable.
